# Supplementary material for: Association between Lifestyle Behaviours and Mental Health of Adolescents: Evidence from the Canadian HBSC Surveys, 2002–2014
Source: Int J Environ Res Public Health. 2022 Jun 4;19(11):6899. doi: 10.3390/ijerph19116899 (PMC9180765; doi:10.3390/ijerph19116899)
Supplement: Supplementary file 1 [file ijerph-19-06899-s001.zip › ijerph-1712681-supplementary.pdf]

Table S1. Sensitivity analysis of associations between physical activity, screen time, and frequent psychosomatic complaints in Canadian adolescents, HBSC 2002-2014.

**Characteristics**	**Overall**	**Boys**	**Girls**
	Adjusted OR (95% CI)	Adjusted OR (95% CI)	Adjusted OR (95% CI)
Physical activity (≥60mins/day)			
≤2 days	1 [Reference]	1 [Reference]	1 [Reference]
3-4 days	0.69 (0.62-0.75)	0.69 (0.59-0.80)	0.69 (0.61-0.78)
5-6 days	0.59 (0.54-0.65)	0.56 (0.48-0.66)	0.62 (0.55-0.70)
7 days	0.68 (0.61-0.75)	0.62 (0.53-0.72)	0.75 (0.65-0.87)
Daily screen time			
≤ 4 hrs/d	1 [Reference]	1 [Reference]	1 [Reference]
4-6 hrs/d	1.20 (1.09-1.31)	1.12 (0.97-1.30)	1.24 (1.10-1.40)
6-9 hrs/d	1.48 (1.35-1.62)	1.40 (1.21-1.61)	1.52 (1.35-1.71)
>9 hrs/d	1.97 (1.80-2.15)	1.75 (1.53-2.00)	2.15 (1.92-2.42)

OR Odds ratio
CI Confidence interval
Models were adjusted for age, body mass index, alcohol consumption, family affluence scale (FAS) score, and survey cycle; the model based on the overall sample is additionally adjusted for gender.
